# Exploring Novel Inhibitory Compounds Against Phosphatase Gamma 2: A Therapeutic Target for Male Contraceptives

**DOI:** 10.3390/cimb47080658

**Published:** 2025-08-15

**Authors:** Hashim M. Aljohani, Bayan T. Bokhari, Alaa M. Saleh, Areej Yahya Alyahyawi, Renad M. Alhamawi, Mariam M. Jaddah, Mohammad A. Alobaidy, Alaa Abdulaziz Eisa

**Affiliations:** 1 Department of Clinical Laboratory Sciences, College of Applied Medical Sciences, Taibah University, Medina 42361, Saudi Arabia; rhamawi@taibahu.edu.sa (R.M.A.); mjidah@taibahu.edu.sa (M.M.J.); 2 Department of Pathology and Laboratory Medicine, College of Medicine, University of Cincinnati, Cincinnati, OH 45221, USA; 3 Department of Clinical Laboratory Sciences, Faculty of Applied Medical Sciences, Umm Al-Qura University, Makkah 24351, Saudi Arabia; btbokhari@uqu.edu.sa (B.T.B.); amsaleh@uqu.edu.sa (A.M.S.); 4 Department of Clinical Laboratory Sciences, College of Applied Medical Sciences, King Saud Bin Abdulaziz University for Health Sciences, Jeddah 11461, Saudi Arabia; yahyawia@ksau-hs.edu.sa; 5 King Abdullah International Medical Research Center, Jeddah 22233, Saudi Arabia; 6 Department of Anatomy, Faculty of Medicine, Umm Al-Qura University, Makkah P.O. Box 7607, Saudi Arabia; maobaidy@uqu.edu.sa

**Keywords:** binding affinity, molecular docking, water swap, MMPBSA/GBSA, contraception

## Abstract

Men have limited options for contraception, despite the widely accepted public health benefits of it, placing the contraceptive burden solely on women. The current study focuses on inhibiting the PP1γ2 enzyme, which plays a role in sperm maturation and motility. The study considered three top compounds based on the findings of molecular docking. The three compounds exhibited a good interaction profile with a binding affinity score of D751-0223 (−8.7 kcal/mol), D751-014 (−8.1 kcal/mol), and N117-0087 (−8 kcal/mol) measured in kcal/mol. Molecular dynamics simulation (MDS) were performed on the PP1γ2–ligand complexes along with the Apo form. The results suggested that all the complexes were stable with no major deviations observed compared to Apo. The average RMSDs for PP1γ2-D751-0223, D751-014, and Apo were 1.27 Å, 1.73 Å, 1.39 Å, and 1.69 Å, respectively. The PP1γ2–ligand complexes were observed with unique salt bridge interactions such as Glu133-Arg137, Asp4-Lys107, Asp188-Arg116, and Glu120-Arg90. The principal component analysis (PCA) findings indicated that every complex had a distinctive motion state. Furthermore, the net MM/PBSA scores for D751-0223, D751-0143, and N117-0087 were −80.01 kcal/mol, −72.18 kcal/mol, and −64.26 kcal/mol, respectively, while the MM/GBSA and MM/PBSA values were −82, −73.07,−67.26 and −80.01, −72.18, −64.26, measured in kcal/mol, respectively. The WaterSwap energy estimation was performed to validate the former technique, and the findings demonstrated that PP1γ2-D751-0223 is a stable complex, with a value of −51.05 kcal/mol. This work provides a baseline to researchers for the identification of novel therapeutic approaches for non-hormonal male contraceptives.

## 1. Introduction

Due to unplanned pregnancies and growing populations worldwide, nations face economic, health, environmental, and social challenges [1]. One of the most crucial elements of family planning and reproductive health involves deciding when to have children. Contraceptives and family planning reached a new phase in 1960 following the advent of Enovid, the very first female hormonal birth control pill that contained mestranol and norethynodrel [2]. Women generally carry the majority of contraceptive responsibility because they have access to a range of hormone-based contraceptive substitutes in different forms [3]. However, more than forty percent (or 100 million) of pregnancies worldwide are unwanted. Approximately 40% of these result in unplanned births, and half end in abortions [4]. According to estimations, a number of women may inevitably die annually as a result of unsafe abortion practices brought on by unplanned pregnancies [5]. To tackle these global issues, there is a dire need to broaden the range of contraceptive alternatives, including male options [6]. For male methods, the unmet demand for contraceptives is significantly higher. There are presently only two types of male contraception: vasectomy, which is safe but not readily reversible, and condoms, which are cheap and efficient for preventing sexually transmitted diseases (STDs), but have a high failure rate of 15–20% in avoiding pregnancies [7]. Nearly 30% of individuals utilize condoms and vasectomy as their primary forms of birth control, considering the lack of other options. Thus, novel male contraceptive methods might decrease unexpected births, meet individuals’ contraceptive needs, and encourage gender equality in terms of the cost of using contraceptives [8].

All eukaryotic cells express Ser/Thr protein phosphatase 1 (PP1), one of the members of the phosphoprotein phosphatase (PPP) class [9]. The primary PP1 isoform in spermatozoa, the sperm-specific gamma 2 variant (PP1c2), is an essential regulator of sperm formation and the initiation and activation of sperm mobility [10]. Several PP1 isoforms (PP1a, PP1b, PP1c1, and PP1c2), having a significant level of sequence similarity (90%), are encoded by three genes [11]. The testis-enriched or sperm-specific PP1 is referred to as PP1γ2, and it is believed to be the primary isoform within mammals that induces PP1 function in spermatozoa [12]. Protein phosphatase inhibitor 2 (PPP1R2, also referred to as I2), protein phosphatase 1 regulating unit 7 (PPP1R7, also called SDS22), and protein phosphatase 1 regulating subunit 11 (PPP1R11, referred to as I3) have been the primary inhibitors that regulate PP1γ2 activity in sperm [10]. Their relationship with PP1γ2 changes as epididymis sperm grow. In short, PP1γ2 has been linked to all three inhibitors in caudal spermatozoa, yet only with I3 in immotile spermatozoa of the caput epididymis. Because of their ability to regulate PP1γ2 activity, these alterations are crucial for the growth of motility [10]. Thus, PP1γ2 has emerged as an attractive drug target for reversible male contraception due to its pivotal role in sperm motility and maturation. Inhibition of this enzyme will stop the biochemical pathways from maturation of sperm by making them non-functional without impairing long-term reproductive health or creating hormonal disorders [13].

**Figure 1:**
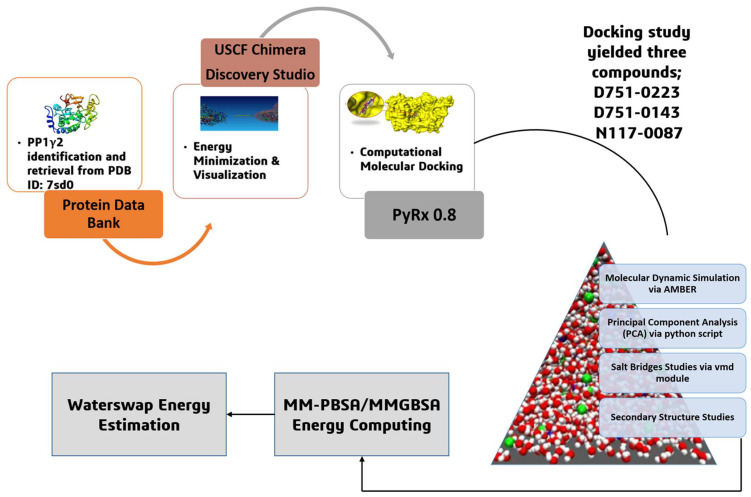
The overall systematic computational study initiated with the retrieval of protein, followed by energy minimization and visualization, molecular docking and molecular dynamic simulation, principal component analysis, salt bridges studies, secondary structure analysis, MM-PBSA/GBSA energy computing, and WaterSwap energy estimation.

Considering the broad field of drug discovery, where chemistry and biology come together, finding and developing an innovative treatment can be a laborious and expensive task [14]. A crucial aspect of modern drug discovery is the powerful and multidisciplinary order of computer-aided drug design, also known as CADD [15]. It discovers and enhances potential drug candidates by fusing biological knowledge with computer systems techniques. The amalgamation of several approaches contributes to CADD’s adaptability and efficacy in the pharmaceutical sector [16]. The broad spectrum of techniques and methods that encourage CADD adds to its scope and adaptability. The efficacy of this field comes from its broad spectrum of methods, such as drug metabolism prediction and structural modeling [17]. For CADD to achieve excellent oral drug-likeness, compounds should ideally minimize violations of criteria such as molecular weight, lipophilicity, hydrogen bond donors, and acceptors. This is achieved by following Lipinski’s rule [18]. Drug development is expedited and enhanced through this systematic integration of CADD methods and adherence to drug-likeness criteria, showing the flexibility and important impact of CADD in a range of areas and the pharmaceutical sector [19]. The present study was conducted to explore novel therapeutic drug molecules against PP1γ2 by using various cheminformatics and bioinformatics techniques. The study utilized different in silico approaches as molecular docking, molecular dynamics simulation, pharmacokinetics profiling, and post-simulation analysis (Figure 1). The study identified three potent inhibitors, but extensive in vivo and in vitro investigation is needed to validate the potential efficacy of the compounds.

## 2. Materials and Methods

### 2.1. Identification, Retrieval, and Preparation of Target Receptor

The three-dimensional (3D) structure of protein phosphatase 1 gamma 2 (PP1γ2) was fetched from the Protein Data Bank (PDB) (https://www.rcsb.org/?ref=nav_home, accessed on 10 February 2025) using ID 7SD0 in UCSF Chimera v1.17. At the time of research work, PDB ID:7SD0 was the latest available structure of PP1γ2. The chain C (sequence length of 292) represents the catalytic subunit of PP1γ2 and was selected for docking investigations despite the structure having multiple chains that form a trimeric complex. All water molecule heteroatoms (apart from any necessary cofactors) and non-target chains were deleted. Following this, polar hydrogens were added to the structure, and Kollman charges were allocated using AutoDock Tools to prepare it for docking. The protein structure was visualized in Discovery Studio Visualizer v2024.

### 2.2. Identification of Binding Pockets

Identifying or predicting the protein’s binding regions is essential for better interaction analysis [20]. The identified active pockets were Arg132, Lys141, Lys147, and Lys150. The active site residues were obtained from [21], who characterized the binding interface using crystallographic data.

### 2.3. Identification and Preparation of Compound Library

A natural product-based compound library consisting of 4561 molecules was retrieved from the ChemDiv database (https://www.chemdiv.com/catalog/focused-and-targeted-libraries/natural-product-based-library/, accessed on 15 February 2025). This readily accessible library consists of small molecules from diverse sources with drug-like characteristics and is inspired by natural products. The library was downloaded in a structure Data file (SDF) format. Ligand parameterization was performed using UCSF Chimera v1.17 before using them in structure-based screening [22]. The ligands were prepared using Dock Prep and the AutoDock Vina plugin. The topology and parameter files were generated via the internally run Parmck tool in UCSF Chimera v.17. The ligand library was then imported to PyRx 0.8, where energy minimization was carried out using Open Babel [23]. Partial charge assignment was performed on the ligands, which were converted into AutoDock Ligand format (PDBQT) [24].

### 2.4. Screening of Compound Library Against the Receptor

A key technique in computer-assisted drug design and structural molecular biology is molecular docking [25]. Ligand–protein docking aims to figure out the most prevalent modes of binding between a ligand and a known protein 3D structure [26]. By using PyRx 0.8, all 4561 compounds were screened against the target receptor [23]. Docking of each ligand with the receptor was performed 50 times. The Vina search space during the screening process was set as follows: Center (X: 100.307 Å, 115.900 Å, and Z: 116.925 Å) and dimensions of 30 Å on the XYZ axes. Thousands of candidates were filtered against the targeted protein based on the lowest binding energy in kcal/mol [27]. To remove undesirable compounds and perform extensive optimization, the compounds with the lowest binding affinity were chosen. In parallel, docking validation was performed by extracting co-crystalized ligands from a PDB ID: 4QDI and re-docked with the mentioned PDB MurF protein using the same procedure discussed above [28]. The validation procedure reported an RMSD value of 0.12 Å, thus illustrating that the protocol is valid. A DSV-formatted Excel document with the binding affinities score and interaction information was retrieved.

### 2.5. Interpretation and Interaction Analysis of Docking Findings

Using AutoDock Vina v4.1 through PyRx 0.8, the docking affinity of compounds was predicted, and only the best binders to the targeted receptor were selected for intermolecular binding conformation and interactions network analysis [29]. Each selected ligand was then subjected to a short-term evaluation where the most optimal binding mode of the compound at the protein active pocket was selected based on the lowest binding energy (in kcal/mol) [30]. Another parameter of evaluation was the enrichment of the ligand interactions network at the active pocket [31]. The best docked complexes, by considering the criteria discussed above, were imported into Discovery Studio Visualizer v2024 for interaction analysis and evaluation [32]. It offered details regarding the complex’s atom type, interactions, forces, and bond lengths [33].

### 2.6. Validating Lipinski Rule of 5 and Pharmacokinetics Assessment

The drug-like characteristics of the best active molecule were studied using the Swiss ADME http://www.swissadme.ch/ (accessed on 10 February 2025) online server accessed on 18 February 2025 [34]. The compound’s standard simplified Molecular Input Line Entry System (SMILES) was given as input [35]. The molecular structure and bioactivity of the drugs with high affinities were predicted using the Swiss ADME. This online server estimates the number of Lipinski’s rule violations about the following parameters: logP, polar surface area, weight, variety of atoms, length of OH, length of rotatable bonds, quantity, enzymes, and nuclear receptors [36]. Moreover, the compounds were evaluated for physicochemical characteristics, medicinal chemistry, lipophilicity, and pharmacokinetic profile.

### 2.7. Molecular Dynamics Simulation

The docked complexes were subjected to molecular dynamics simulation using the AMBER v20 software. It offers information about the accuracy of the intermolecular interactions and predicts how proteins will respond when the compounds interact with them in dynamic environments [36]. The docked complex was provided as an input in PDB format for this analysis [31]. The MD simulations were performed in three steps: (1) system preparation, (2) preprocessing step, and (3) production run [37]. The AMBERv20 software suite was employed to perform MD simulations using the ff19SB force field for the protein and the GAFF2 [38] force field for ligand parameters, which were produced using Antechamber [39]. An octahedral box of TIP3P water molecules was used to solvate the protein–ligand complexes, keeping a padding distance of 12 Å. To neutralize the system, Na+ counter ions were added [40]. The energy minimization was carried out in two stages: steepest descent and conjugate gradient algorithms for a total of 3000 rounds. During the process, the restraining force of 100 kcal/mol on hydrogen atoms, water, and sodium ions was applied, followed by a 5 kcal/mol energy constraint in the second round. Firstly, the minimization of ions and water with protein and ligands was carried out, followed by the minimization of the whole system [41]. Each system was then heated up to 310 K with a restraint of 5 kcal/mol on the carbon alpha atoms. The canonical ensemble was applied during the heating step. Afterward, equilibration was performed for 1 ns using the NPT ensemble with no restraints. The MD simulation was run for 200 ns, and the trajectories were analyzed by the xmgrace v5.1 module [42]. The SHAKE algorithm was used to remove stretching of fast bond motions [43]. The particle mesh Ewald method was employed to approximate long-range interactions [44]. The CPPTRAJ module of AMBER was used for MD trajectory analyses [45].

### 2.8. Salt Bridges (SS) Analysis

Protein–ligand complexes can be stabilized by non-covalent salt bridges (SS), which are effective bonds in drug development [46]. For instance, PP1γ2 can be inhibited by small compounds, where SS can play a crucial role. The SS analysis was performed by the Visual Molecular Dynamics (VMD) software v1.93, developed by University of Illinois at Urbana, CA, USA [47]. The distance cut-off set during this analysis was 3.0.

### 2.9. Principal Component Analysis (PCA)

PCA is an approach that transforms coordinated observations into orthogonal vectors called principal components. PCs can be explained as variance in data. PCA helps reduce the dataset’s complexity, promoting understanding while minimizing information loss [48]. PCA is widely used in downstream MD analysis for identifying dominant motions and reducing dimensionality. The collective motions of atoms are represented by the PC1, PC2, PC3, and PC4 [36]. The AMBER CPPTRAJ program was utilized for plotting PCA.

### 2.10. Secondary Structure Analysis

A secondary structure analysis was performed to assess the structural changes and patterns in the protein structure upon binding to the ligand [49]. The proportion of secondary structure content in a protein is a crucial statistic in the investigation of changes in structural behavior [50]. The secondary structure analysis was carried out using the AMBER CPPTRAJ module.

### 2.11. MMPB/GBSA Calculations

The most efficient post-MD technique to calculate the binding free energy of a system (ligand–receptor) is the Molecular Mechanics/Generalized Born Surface Area (MMGB/PBSA) [51]. The analysis was performed using the AMBER MMPBSA.py module. The MMPB/GBSA uses the following equations to carry out the calculations [52]:

∆Gbind = G complex − Gprotein − Gligand(1)

∆Gbind = ∆Ggas + ∆Gsol − T∆S(2)

∆Ggas = Bond + Angle + Dihed + EEL + VDWAAL(3)

∆Gsol = ∆EGB + ∆ESURF

where binding net energy is denoted with ∆Gbind (Equation (1)), calculated by utilizing Equation (2). T∆S represents the variation in conformational entropy. ∆Ggas denotes the sum of the bond angle, Van der Waals, and electrostatic part of the internal energy (Equation (3)), while the ∆Gsol, called salvation energy, is the combination of polar solvation energy (∆EGB) and non-polar solvation energy (∆ESURF). The MMPB/GBSA analysis was performed on 5000 frames picked at regular intervals from simulation trajectories.

### 2.12. WaterSwap Energy Estimation

The complicated nature of enzyme–water and drug–water interactions is usually not taken into consideration by the MM/GBPBSA approach, which uses an implicit water system and takes snapshots at regular times of MD simulations [53]. On the other hand, WaterSwap addressed the limitations of MM/GBPBSA by employing an explicit water model. A computational tool called WaterSwap can calculate the absolute binding free energy by swapping equal volume water in the protein binding pocket with a ligand [54]. Three algorithms were used for this calculation: free-energy perturbation (FEP), Bennett’s acceptance ratio (BAR), and thermodynamic integration (TI) [55]. An energy difference of 1 kcal/mol among the mentioned three algorithms indicates that the system’s energy converged well.

## 3. Results

### 3.1. Identification, Retrieval, and Preparation of Male Contraceptives with the Main PP1γ2

**Figure 2:**
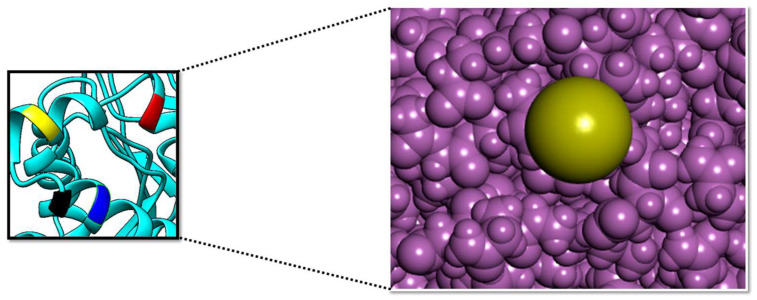
The surface of the PP1γ2 active pockets (**right side**); the surface of the Arg132 (yellow), Lys141 (red), Lys147 (black), and Lys150 (blue) active pockets (**left side**). The purple color shows the active site surface in close view.

PP1γ2 is testis-specific and helps in sperm maturation. Inhibiting this is a promising strategy for non-hormonal male contraceptives [56]. The crustal structure of the target enzyme was downloaded from PDB via ID: 7sd0. The target protein is accessible at 2.95 Å by the electron microscopy method. The protein has Global Symmetry: Asymmetric-C1 and Global Stoichiometry: Hetero 3-mer—A1B1C1. The structure was energy-minimized and subsequently visualized by Discovery Studio [32]. The crystal structure, along with the active pockets labeled, is depicted in Figure 2.

### 3.2. Molecular Docking Analysis

In silico molecular docking was carried out using AutoDock Vina to analyze the molecular interactions and the binding affinity of the protein with the Natural Product-Based Library consisting of 4561 compounds. To identify the best binding from the drug library, target-based virtual screening was performed [57]. The docking findings yielded the top 10 compounds based on their docking score, ranging from −8.7 to −7 kcal/mol. Binding mode and interaction analysis were performed on the first three hits that were considered to be the best binding compounds based on their lower binding affinity value. The top 10 compounds selected are given in Table 1, presenting detailed information about the compound’s chemical name, binding score, chemical structure, and H-bond interactions involved.

### 3.3. Docking Analysis and Interpretation of Selected Hits

**Figure 3:**
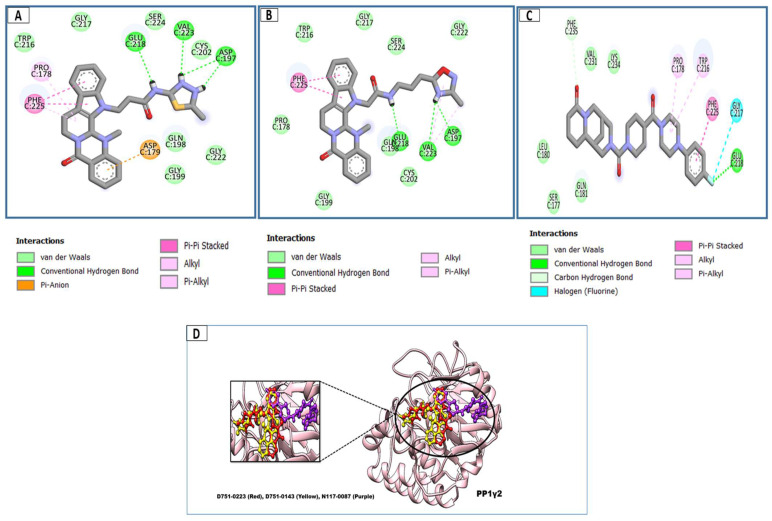
The two-dimensional (2D) and three-dimensional (3D) images of the top three hits: D751-0223 (**A**), D751-0143 (**B**), and N117-0087 (**C**), with a close-up view of the binding poses and interactions (**D**).

Each of the three compounds is strongly attached to the binding pocket of PP1γ2. The top-1 hit compound (D751-0223) is 2-methyl-5-(3-(14-methyl-5-oxo-7,8,13b,14-tetrahydroindolo[2″,3′:3,4]pyrido[2,1-b]quinazolin-13(5H)-yl)propanamido)-1,3,4-thiadiazole-3,4-diium, where the compound is strongly bound to three hydrogen atoms as VAL 223, ASP197, and GLU218. Besides h-bonding, several van der Waals interactions were noted: TRP 216, CYS202, GLY222, GLY199, and GLN198 (Figure 3A). The top hit (D751-0143) is N-(3-(3-methyl-1,2,4-oxadiazolidin-5-yl)propyl)-2-(14-methyl-5-oxo-7,8,13b,14-tetrahydroindolo[2′,3′:3,4]pyrido[2,1-b]quinazolin-13(5H)-yl)acetamide, showing three h-bonds, Glu218, Asp197, and Val223, while the van der Waals interactions formed were Trp216, Gly217, Ser224, Gly222, and Pro178 (Figure 3B). (N117-0087) is 3-(4-(4-(4-fluorophenyl) piperazine-1-carbonyl) piperidine-1-carbonyl) octahydro-1H-1, 5-methanopyrido [1, 2-a] [1, 5] diazocin-8(2H), with one h-bond observed with residue GLU218. On the other hand, the compound formed van der Waals interactions with Phe235, Lys237, Val231, Leu180, Ser177, and Gln181, along with other alkyl and pi-alkyl interactions (Figure 3C). The top three hits were found to possess good binding affinity and appropriate interactions with the residues of the enzyme’s active site. Hence, the three hits seem to be novel inhibitors of PP1γ2. The intermolecular binding mode of the selected compounds with the PP1γ2 enzyme can be seen in Figure 3D.

### 3.4. Lipinski Rule of 5 and Pharmacokinetics Characteristics

Swiss ADME assessed the criteria of Lipinski’s rule and other parameters of drug-likeness, such as mass; logP; polar surface area; range of atoms; range of O, N, or NH; hydrogen bond donors; total number of hydrogen bond recipients; and range of rotatable bonds, as shown in Table S1. The top three hits met the Lipinski Rule 5 criteria and were deemed drug-like. Moreover, the physiochemical features, pharmacokinetics, and medicinal chemistry were within the acceptable range. The compounds were processed for further computational screening through MD simulation.

### 3.5. Molecular Dynamic Simulation of PP1γ2–Ligand Complexes and Apo

**Figure 4:**
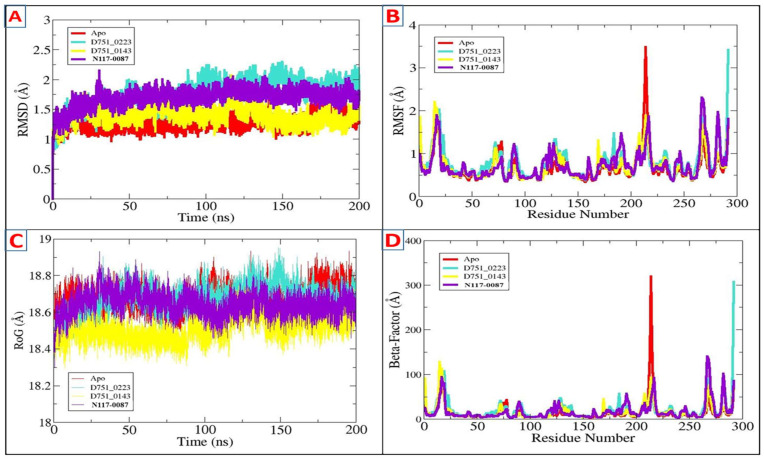
Findings of MDs through AMBER xmgrace module. (**A**) RMSD, (**B**) RMSF, (**C**) RoG, and (**D**) beta factor for D751-0223 (cyan), D751-0143 (yellow), and N117-0087 (purple) in comparison with Apo (Red).

The stability and dynamics of PP1γ2-compound complexes were studied using a 200 ns molecular simulation. To determine the intermolecular strength of interaction and steady dynamics of complexes, multiple statistical analyses were carried out from the simulation trajectories, which include the radius of gyration (RoG), RMSD, root mean square fluctuation (RMSF), beta factor, and solvent accessible surface area (SASA) [58]. The lower RMSD shows the greater stability of the receptor protein upon ligand binding and vice versa [59]. The RMSD (Figure 4A) of all three complexes showed that they attained stability at the start of the simulation period, with the ligand-bound systems exhibiting decreased deviations compared to Apo, indicating better stability upon ligand binding. The average RMSD scores for D751-0223, D751-0143, N117-0087, and Apo are 1.27 Å, 1.73 Å, 1.39 Å, and 1.69 Å, respectively. The RMSF of the complexes was evaluated afterward to identify residue-specific fluctuations in the compound’s presence at the enzyme’s active region. The majority of the receptor residues are in a good stable range, with a mean RMSF < 2 Å, as shown in Table 2. Certain variations that lead to a higher RMSF are driven by specific residues with notable fluctuations around 200 in the Apo. The fluctuations then decreased upon ligand binding (Figure 4B). The analysis of the beta factor validated the flexibility seen in the RMSF plot, representing greater values of Apo at 200 residues. The average beta factor scores were D751-0223 (14.75 Å), D751-0143 (20.29 Å), N117-0087 (15.56 Å), and Apo (17.29 Å). The PP1γ2 rigidity and compactness during the 200 ns simulation period were then assessed by the radius of the gyration plot. A higher ROG score represents reduced rigidity, while a lower value suggests a more compact structure of PP1γ2 (Figure 4C). The D751-0223, D751-0143, and N117-0087 showed lower RoG compared to Apo, suggesting more compactness upon ligand binding. The RoG values for the top three hits are mentioned in Table 2. Similarly, the beta factor analysis was performed, which confirms the RMSF analysis and validates that there are no major residue-wise fluctuations (Figure 4D).

**Figure 5:**
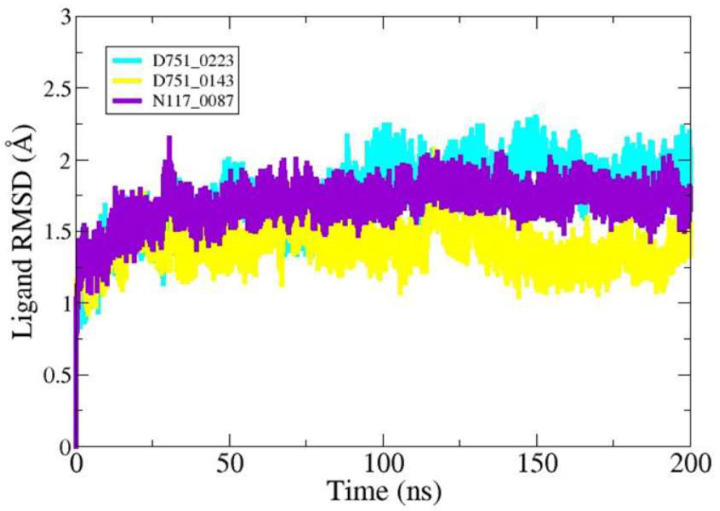
Compounds’ RMSD over simulation time.

**Figure 6:**
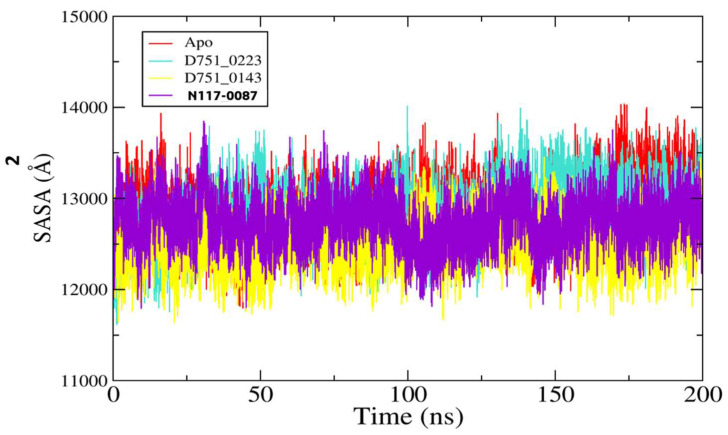
The analysis of SASA for D751-0223 (cyan), D751-0143 (yellow), and N117-0087 (purple) in comparison to Apo (red).

Further, ligand RMSD analysis was performed in order to investigate ligand dynamics stability over the course of simulation time (Figure 5). As can be seen in the figure, the RMSD plot of each ligand shows constant stability with no observed major fluctuations. The minor fluctuations noticed were due to flexible loops in the protein structure, which naturally allow the protein to accommodate the bounded ligand. The mean RMSDs of D751-0223 (cyan), D751-0143 (yellow), and N117-0087 (purple) are 1.73 Å, 1.39 Å, and 1.69 Å, respectively. The analysis of solvent-accessible surface area was conducted to assess the surface region of PP1γ2 exposed to solvents such as water [58]. D751-0223-PP1γ2 complex showed a good SASA score with higher exposure to a solvent, indicating better stability in its structure. The maximum SASAs for the top three PP1γ2 complexes, along with Apo, were 140.39 Å^2^, 14,014.4 Å^2^, 136,462.7 Å^2^, and 13,850.9 Å^2^. The SASAs indicate no major water molecule concentration shift, indicating the stable equilibrium of the protein–ligand complexes (Figure 6).

### 3.6. Salt Bridge Studies: (SB)

Salt bridges are formed when proteins containing oppositely charged residues are sufficiently connected to attract each other via electrostatic attraction [60]. While they are unlikely to naturally increase a protein’s free energy of unfolding, they can contribute to the protein structure and the unique characteristics of interactions. Table 3 offers a detailed overview of the SB interactions of complexes and the Apo form. SB non-covalent interactions that combine electrostatic interactions between charged residues are typically positive and negative [61]. The greatest number of salt bridges in proteins is found between the negatively charged acidic Asp and Glu residues and the positively charged amino acid residues, such as Lys or Arg [62]. The permissible distance for SB is <4 Å [46]. The complexes and the Apo form that shared common SB interactions are Glu96-Arg137, Glu178-Arg182, Glu28-Arg9, and Asp132-Arg136. These interactions were redundant and repetitive, suggesting their role in protein stability and functionality. The unique SBs observed are Glu133-Arg137 (D751-0223), Asp4-Lys107 (D751-0223 and Apo), Asp188-Arg116 (D751-0143), and Glu120-Arg90 (N117-0087 and Apo), as shown in Figures S1 and S2. The unique SBs formed as a result of conformational variations upon ligand binding to the protein, supporting the stability of complexes in specified functional conditions. The complexes remained stable during the 200ns simulation since the SB interactions length was under the permissible range (<4 Å).

### 3.7. Principal Component Analysis (PCA)

**Figure 7:**
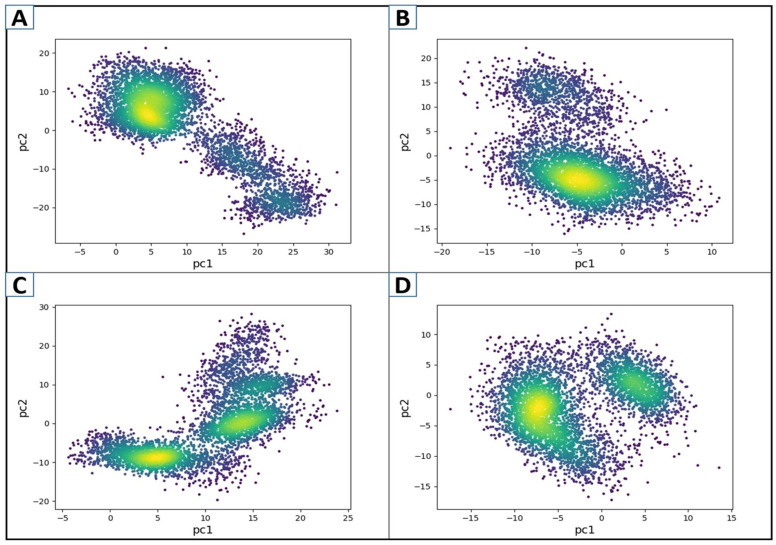
The PCA plots of the first two components, PC1 and PC2, for D751-0223-PP1γ2 (**A**), D751-0143-PP1γ2 (**B**), N117-0087-PP1γ2 (**C**), and Apo form (**D**). Color gradient represents simulation frames (from blue to yellow to red).

The structural and energy data from MD simulation on protein–ligand complexes and Apo proteins are evaluated utilizing principal component analysis (PCA) [63]. The coordinate covariance matrix generated from the 200 ns MD simulation of hit compound complexes was subjected to PCA to capture the most noticeable motions over the MD simulation [64]. The PCA was plotted for the first two components, such as PC1 and PC2, capturing the majority of the data. Here, the PCA for D751-0223-PP1γ2, D751-0143-PP1γ2, and N117-0087-PP1γ2 complexes was performed along with Apo (unbound state). The periodic changes were noticed by the color gradient changing from purple to yellow. Every complex had a distinctive motion style, according to PCA. With blue dots at the start and purple at the end of the spectrum, the motion of the D751-0143, N117-0087, and Apo forms was relatively scattered (Figure 7). In contrast, the D751-0223-PP1γ2 exhibited a compact and clustered form of motions covering the majority of conformational data in PC1 compared to other complexes. On the contrary, the N117-0087-PP1γ2 (C) and Apo form (D) exhibited two distinct clusters, which may show transitions between multiple conformational states, suggesting less stable binding. The PCA for PP1γ2–ligand complexes, along with Apo, is depicted in Figure 7.

### 3.8. Secondary Structure Studies (SS)

**Figure 8:**
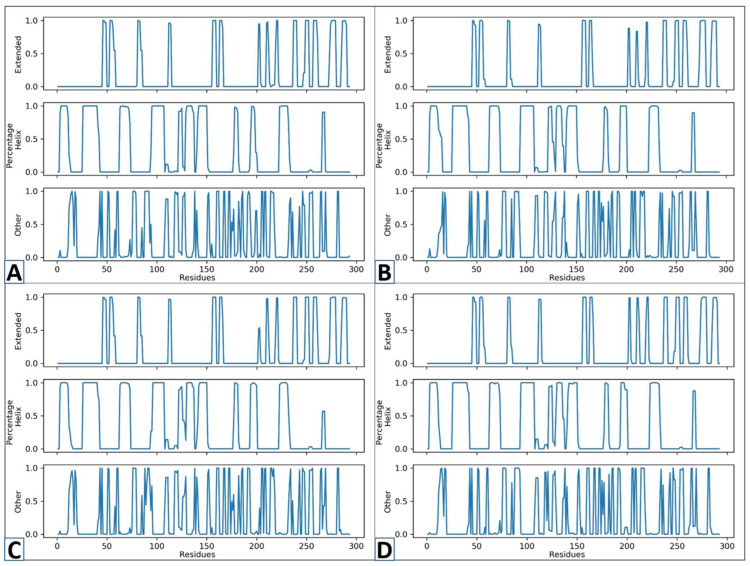
The secondary structure studies of D751-0223 (**A**), D751-0143 (**B**), N117-0087 (**C**), and Apo (**D**) from.

To determine the changes in secondary structures induced by the binding of the specified compound, an analysis of secondary structure was carried out on the complexes and Apo form [65]. The percentage of secondary structure concentration in a protein is an important variable in the study of structural behavior changes [50]. Figure 8 depicts the SS changes across their different residues. The y-axis in the figure represents the frequency of various structural conditions, while the x-axis shows the residue numbers. The three rows in each figure of complexes show different significances. The row named “Extended” designates extended structures and beta-sheets, while the “percentage” indicates the alpha-helical sites of the protein. Furthermore, the “other” sections in the figure specify coils and turns. The novel identified compounds’ secondary structure percentage stayed mostly unchanged. However, it seems that N117-0087 showed a consistent SS. With extended sites and fewer unstructured residues. The portion of the alpha helix and beta sheets was largely unchanged, suggesting that ligand binding did not cause significant changes in the structure. This shows a non-disruptive interaction of the protein and ligand.

### 3.9. MM/GBSA and MM/PBSA Calculations

In computer-assisted drug design, the MM/GBSA and MM/PBSA techniques are frequently utilized to evaluate a drug’s binding affinity towards a specific biological macromolecule [66]. One of the benefits of these techniques is that they require fewer computing resources than chemical binding free-energy techniques [67]. The strategies additionally act as endpoint ways of evaluating the various binding energies resulting from the drugs’ interaction with the target. All three virtually identified hit compounds exhibited strong Van der Waals and electrostatic energy contributions. The van der Waals contributed significantly to all compounds, followed by electrostatic energy. These two types of energy combine to form the gas-phase energy of the compounds. The MMGBSA/PBSA values are given in detail in Table 4. The primary field of difference between the two methods is how they handle solvation effects, yet both calculate binding free energy using molecular mechanics energies [36]. While MM/PBSA utilizes the Poisson–Boltzmann (PB) model, which solves the PB equation to produce more precise electrostatic solvation energy values, MM/GBSA uses the Generalized Born (GB) model, which corresponds to the solvent environment more quickly but less correctly. While MM/GBSA delivers far quicker processing, MM/PBSA is typically thought to be more physically demanding. Combining the two enables a complementary assessment of binding energetics [68]. The net MM/GBSA energy for D751-0223 (−82 kcal/mol), D751-0143 (−73.07 kcal/mol), and N117-0087 (−67.26 kcal/mol). Furthermore, the net MP/GBSA scores for D751-0223, D751-0143, and N117-0087 were −80.01 kcal/mol, −72.18 kcal/mol, and −64.26 kcal/mol, respectively. According to the findings, each of the three compounds achieved a stable phase within the enzyme’s region and was bound by a strong system of chemical bonds.

### 3.10. WaterSwap Energy Estimation

Protein–water, ligand–water, and protein–water–ligand interaction characteristics are considered by the precise solvation mechanism utilized by WaterSwap [69]. Since the MMGB/PBSA does not have this data, it cannot be used to precisely predict where water molecules might work in interactions between proteins and ligands. This is particularly important in cases where water molecules are employed to link the ligand to the receptor. The WaterSwap method is vital to figuring out total binding free energy and has been successfully utilized in a variety of biological settings [54]. After 1000 frames, the WaterSwap energies for each PL complex exhibited a considerable convergence. Furthermore, every number suggested that the intermolecular docked configuration was stable. Three algorithms, such as Bennet’s, free-energy perturbation (FEP), and thermodynamic integration (TI), were used to calculate the net mean (Table 5). The findings suggest that PP1γ2-D751-0223 ranked first, with a value of −51.05, followed by PP1γ2-D751-0143 (−47.21) and PP1γ2-N117-0087 (−37.2).

## 4. Discussion

The world’s population, which has been increasing at an alarming pace, is estimated to exceed 9.8 billion people by 2050 [12]. Despite the provision of contraceptive methods, nearly 40% of pregnancies globally were unplanned between 2010 and 2014 [12]. Male contraceptive options are more limited and less frequently used than female options [70]. Hormonal control has remained the main objective of modern male contraceptive technology so far; however, the pharmaceutical sector stopped much of its efforts in this area after receiving reports of serious adverse effects [71].

The current study was conducted to develop non-hormonal male contraceptives by inhibiting PP1γ2 to avoid systemic adverse effects. In this research, a systematic in silico investigation was performed to target the enzyme PP1γ2, which plays a key role in sperm maturation. A compound library called the Natural Product-Based Library, consisting of 4561 compounds, was used against the target receptor PP1γ2. Extensive virtual screening was carried out to identify potential inhibitors of PP1γ2. The study selected the top 10 hits based on their binding affinities, indicating strong interactions with the target in a static environment. For further analysis, the three most promising compounds were chosen as lead compounds. These compounds—D751-0223, D751-0143, and N117-0087—exhibited binding affinities of −8.7 kcal/mol, −8.1 kcal/mol, and −8.0 kcal/mol, respectively. Molecular dynamics simulation was then performed in three phases: (i) system preparation, (ii) pre-processing, and (iii) the production run of these three compound-PP1γ2 complexes, along with the Apo form. A detailed analysis of the MD simulation trajectories included the RMSF, RMSD, RoG, beta factor, and SASA.

The in silico study suggested that the PP1γ2–ligand complexes showed improved overall stability and compactness over the simulation run compared to the Apo form (the unbound state). This suggested that the protein achieved stability with less deviation upon binding to ligands. To further proceed with the study, the salt bridge analysis of three complexes showed unique interactions. such as Glu133-Arg137 (D751-0223), Asp4-Lys107 (D751-0223 and Apo), Asp188-Arg116 (D751-0143), and Glu120-Arg90 (N117-0087 and Apo). Unique SBs were created due to conformational variations upon ligand binding to protein, aiding in the stability of the PP1γ2 complexes identified. The PCA demonstrated that each PP1γ2-complex had a distinct motion style, according to PCA. The MM/PBSA and MM/GBSA binding free energy classified the compounds as follows: D751-0223 > D751-0143 > N117-0087, with free-energy scores of −93.05 kcal/mol, −88 kcal/mol, and −80.75 kcal/mol.

A study was conducted by [72] to disrupt sperm motility by inhibiting an enzyme called sperm hyaluronidase. This study involved both experimental and computational analysis and utilized bioactive compounds from natural sources with contraceptive properties. Natural bioactive compounds with contraceptive potential were great substitutes for current hormonal methods of contraception. Reversible and non-hormonal contraceptives for men can be developed from natural non-hormonal compounds that inhibit the enzyme sperm hyaluronidase. The male contraceptive characteristics of the plant “Aegle marmelos Linn” leaf extracts were evaluated using the in vitro inhibition method along with computer-assisted methods. In silico approaches such as molecular docking, molecular dynamics, non-covalent interaction evaluation, molecular mechanics, and Poisson–Boltzmann surface area were used to identify the interaction pattern of marmin, aegeline, and marmenol on hyaluronidases. The findings from the use of computer-assisted sperm analysis and the in vitro hyaluronidase inhibition assay suggest that the usage of leaf extracts limits the enzymatic activity of hyaluronidase and decreases sperm counts. The findings from the in silico approach suggest that phytocompounds such as aegeline and marmin have the potential to inhibit the enzyme sperm hyaluronidase.

Compared to standard combinatorial chemistry, CADD employs a considerably more focused search, which may boost the hit rate for novel therapeutic molecules [73]. Apart from elucidating the molecular foundations of therapeutic actions, it also seeks to predict potential compounds that could enhance efficiency [74]. Three main objectives are frequently achieved by CADD in drug discovery: (1) filtering massive compound libraries into reduced sets of suggested active compounds that can be evaluated through experiments; (2) contributing to the optimization of hits, whether the objective is to enhance their affinity or just their features of drug metabolism and pharmacokinetics (DMPK), including absorption, distribution, metabolism, excretion, and possibility of toxicity (ADMET); and (3) developing novel drugs by either putting together pieces into distinct chemotypes or “developing” and setting up molecules one functional group at a time [75]. The study identified three novel drugs targeting thePP1γ2 with promising computational findings, but further experimental validation needs to be conducted to improve their efficiency. Though the CADD results presented in this work are interesting, they have several limitations. CADD relies on computational models, which are usually simplifications of the complex biological reality and thus cannot represent all the microscopic events in real time. Thus, the models may result in inaccurate binding affinity predictions, off-target effects, or pharmacokinetics. Also, the findings of the study might not be able to fully capture the complexity of system biology, polypharmacology, and multi-target effects.

## 5. Conclusions

To sum up, this in silico study employed molecular docking and molecular dynamics modeling to investigate how PP1γ2 binds to multiple ligands. The top three compounds with the best binding energy score were selected as drug candidates. The protein–ligand (PL) complexes’ flexibility and stability at the molecular level in a dynamic environment were shown by a 200 ns molecular dynamics simulation. The best compounds identified (D751-0223, D751-0143, and N117-0087) could potentially be the most effective against the target protein based on the latest research. However, further extensive investigation (including both in vitro and in vivo testing) is required to confirm their accuracy and performance as successful therapeutic options.

## Figures and Tables

**Table 1:** The top 10 hits with their IDs, chemical name and structure, binding affinities score, and associated hydrogen bond interactions.

S.No	Compound ID	Chemical Name and Structure	Binding Affinity	H-Bonds Interaction	Hydrophobic Interactions
1.	D751-0223	2-methyl-5-(3-(14-methyl-5-oxo-7,8,13b,14-tetrahydroindolo[2′,3′:3,4]pyrido[2,1-b]quinazolin-13(5H)-yl)propanamido)-1,3,4-thiadiazole-3,4-diium 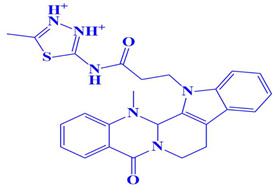	−8.7 kcal/mol	Glu218, Val223, Asp197	Gly217, Trp216, Ser224, Cys202, Gln198, Gly199, Gly222
2.	D751-0143	N-(3-(3-methyl-1,2,4-oxadiazolidin-5-yl)propyl)-2-(14-methyl-5-oxo-7,8,13b,14-tetrahydroindolo[2′,3′:3,4]pyrido[2,1-b]quinazolin-13(5H)-yl)acetamide 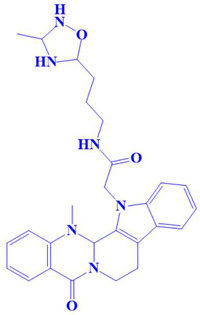	−8.1 kcal/mol	Glu218, Asp197, Val223	Trp216, Gly217, Ser224, Gly222, Pro178, Gln198, Cys202, Gly199
3.	N117-0087	3-(4-(4-(4-fluorophenyl)piperazine-1-carbonyl)piperidine-1-carbonyl)octahydro-1H-1,5-methanopyrido[1,2-a][1,5]diazocin-8(2H)-one 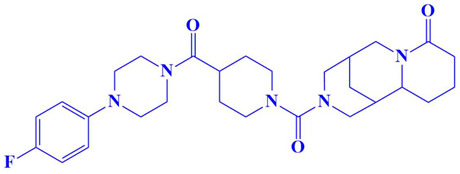	−8 kcal/mol	Glu218	Phe235, Val231, Lys234, Leu180, Ser177, Gln181,
4.	N117-0096	3-(4-([1,4′-bipiperidine]-1′-carbonyl)piperidine-1-carbonyl)octahydro-1H-1,5-methanopyrido[1,2-a][1,5]diazocin-8(2H)-one 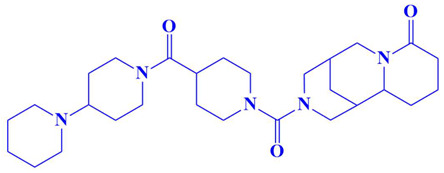	−8 kcal/mol	Asp194, Leu210	Pro196, Th193, Leu200, Glu199, Arg188, Gln185, Val195, Met190, Ile189, Asp179
5.	D751-0222	2-(3-(14-methyl-5-oxo-7,8,13b,14-tetrahydroindolo[2′,3′:3,4]pyrido[2,1-b]quinazolin-13(5H)-yl)propanamido)pyridin-1-ium 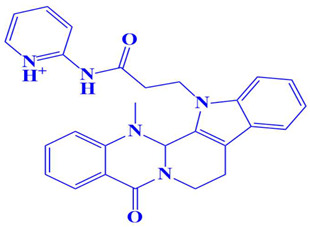	−7.9 kcal/mol	Asp197	Trp216, Gly217, Gly199, Glu218, Gln198, Ser224, Val223, Gly222
6.	D751-0254	N-(2-(3-methyl-1,2,4-oxadiazolidin-5-yl)ethyl)-3-(14-methyl-5-oxo-7,8,13b,14-tetrahydroindolo[2′,3′:3,4]pyrido[2,1-b]quinazolin-13(5H)-yl)propanamide 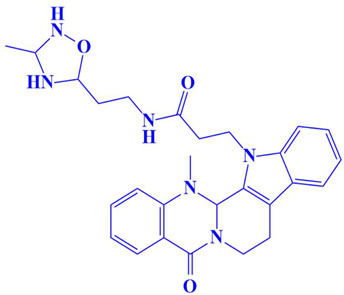	−7.9 kcal/mol	Glu218, Asp197	Gly217, Ser224, Glu222, Gln198, Val223, Cyc202
7.	D751-0214	3-(14-methyl-5-oxo-7,8,13b,14-tetrahydroindolo[2′,3′:3,4]pyrido[2,1-b]quinazolin-13(5H)-yl)-N-(2-morpholinoethyl)propanamide 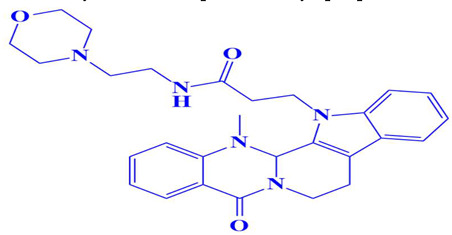	−7.7 kcal/mol	Glu218	Trp216, Gly217, Asp179, Glu199, Gln198, Asp197, Ser224, Val223, Gly222
8.	CM3007-1542	(4-fluoro-1-(mesitylsulfonyl)pyrrolidin-2-yl)(1H-indol-1-yl)methanone 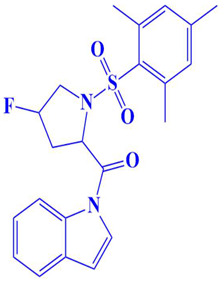	−7.5 kcal/mol	**-**	Trp216, Glu218, Gly217, Ser224, Val223, Asp197, Gln198, Gly199, Leu200, Ser177, Pro178,
9.	CM4579-5752	3-((4,4-difluoro-1-(5-(4-fluorophenyl)-2,5-dihydro-1H-pyrazole-3-carbonyl)pyrrolidin-2-yl)methoxy)pyridin-1-ium 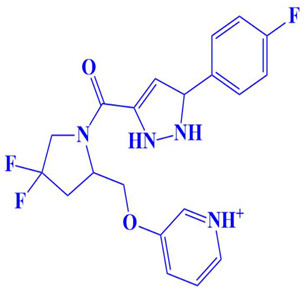	−7 kcal/mol	Trp216, Gln181	Lys234, Phe235, Ser177, Leu176
10	D751-0108	13-(2-((3-(2,3-dihydro-1H-imidazol-1-yl)propyl)amino)-2-oxoethyl)-14-methyl-8,13-dihydro-7H-indolo[2′,3′:3,4]pyrido[2,1-b]quinazolin-6,14-diium-5-olate 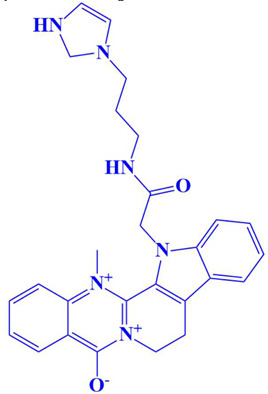	−7 kcal/mol	Arg188	Thr193, Met190, Ile189, Leu201, Gln198, Asp179, Gln185

**Table 2:** The simulation values for the top three complexes, D751-0223, D751-0143, and N117-0087, in comparison to Apo.

RMSD			
Complexes	Minimum	Maximum	Mean
D751-0223	-	1.69 Å	1.27 Å
D751-0143	-	2.30 Å	1.73 Å
N117-0087	-	2.07 Å	1.39 Å
Apo	-	2.14 Å	1.69 Å
**RMSF**			
**Complexes**	**Minimum**	**Maximum**	**Mean**
D751-0223	0.34 Å	3.48 Å	0.66 Å
D751-0143	0.37 Å	3.41 Å	0.80 Å
N117-0087	0.36 Å	2.20 Å	0.70 Å
Apo	0.35 Å	2.30 Å	0.73 Å
**Beta Factor**			
**Complexes**	**Minimum**	**Maximum**	**Mean**
D751-0223	3.08 Å	319.27 Å	14.75 Å
D751-0143	3.65 Å	307.12 Å	20.29 Å
N117-0087	3.44 Å	127.79 Å	15.56 Å
Apo	3.29 Å	139.82 Å	17.29 Å
**RoG**			
**Complexes**	**Minimum**	**Maximum**	**Mean**
D751-0223	18.43 Å	18.93 Å	18.67 Å
D751-0143	18.34 Å	18.95 Å	18.67 Å
N117-0087	18.29 Å	18.74 Å	18.50 Å
Apo	18.29 Å	18.93 Å	18.64 Å
**SASA**			
**Complexes**	**Minimum**	**Maximum**	**Mean**
D751-0223	117.93 Å^2^	140.39 Å^2^	12,890.7 Å^2^
D751-0143	11,612.1 Å^2^	14,014.4 Å^2^	12,854.5 Å^2^
N117-0087	11,639.3 Å^2^	136,462.7 Å^2^	12,541.8 Å^2^
Apo	11,453.5 Å^2^	13,850.9 Å^2^	12,743.7 Å^2^

**Table 3:** The salt bridge interactions for D751-0223, D751-0143, N117-0087, and Apo. SB (Salt bridges).

Complexes	Salt Bridges Interaction	Unique SB Interactions
D751-0223	Glu133-Arg137, Asp202-Arg240, Asp202-Arg215, Glu178-Arg182, Asp202-Arg215, Glu178-Arg182, Glu38-Arg37, Glu224-Lys228, Glu178-Arg182, Glu96-Arg137, Glu178-Arg182, Glu28-Arg9, Glu28-Arg9, Asp65-Arg68, Glu71-Arg14, Glu26-Lys141, Asp86-Arg90, Glu38-Lys35, Glu26-Arg30, Glu48-Arg181, Glu48-Arg181, Asp148-Arg37, Asp4-Lys107, Asp132-Lys135, Glu178-Arg181, Glu38-Lys35, Asp132-Lys135.	Glu133-Arg137 Asp4-Lys107
D751-0143	Asp214-Arg215, Asp148-ly144, Asp202-Arg215, Asp132-Arg136, Asp160-Lys162, Glu178-Arg182, Glu96-Arg137, Asp206-Lys205, Asp160-Lys162, Glu28-Arg9, Glu26-Lys141, Asp86-Arg90, Glu26-Lys141, Glu26-Arg30, Asp188-Arg116, Glu48-Arg181, Glu26-Arg30, Glu133-Arg136, Asp236-Lys162, Asp204-Lys205, Glu133-Arg136, Asp148-Arg37, Glu133-Lys92, Glu71-Arg14, Glu178-Arg181, Asp236-Lys162, Glu178-Arg181, Glu161-Lys162, Glu178-Arg181.	Asp188-Arg116
N117-0087	Glu12-Lys20, Glu28-Arg9, Glu96-Arg137, Asp148-Lys144, Glu178-Arg182, Asp202-Arg240, Asp132-Arg136, Glu178-Arg182, Glu224-Lys228, Glu178-Arg182, Glu96-Arg137, Asp132-Arg136, Glu28-Arg9, Glu26, Lys141, Glu71-Arg14, Glu71-Arg14, Asp86-Arg90, Glu71-Arg14, Asp86-Arg90, Glu38-Lys35, Glu120-Arg90, Glu133-Arg136, Glu48-Arg181, Asp234-Lys162, Asp236-Lys162, Asp132-Lys135, Glu38-Lys35, Glu26-Arg30, Glu178, Arg181	Glu120-Arg90
Apo	Glu12-Lys20, Asp148-Lys144, Glu96-Arg137, Glu28-Arg9, Asp202-Arg240, Glu38-Arg37, Glu96-Arg137, Asp132-Arg136, Glu96-Arg137, Asp132-Arg136, Glu96-Arg137, Glu28-Arg9, Asp206-Lys205, Asp65-Arg68, Glu71-Arg14, Asp86-Arg90, Glu26-Arg30, Glu48-Arg181, Glu26-Arg30, Glu48-Arg181, Glu26-Arg30, Glu120-Arg90, Glu48-Arg181, Asp148-Arg37, Glu178-Arg181, Asp4-Lys107, Asp236-Lys162, Asp148-Arg37, Glu38-Lys35, Asp234-Lys162, Glu178-Arg181.	Asp4-Lys107 Glu120-Arg90

**Table 4:** The top compounds’ (D751-0223, D751-0143, and N117-0087) MM/GBPBSA binding free-energy investigation. The units of measurement are kcal/mol.

Method	Energy Section	D751-0223	D751-0143	N117-0087
MM/GBSA	Van der Waals Energy	−75.04	−72.36	−65.87
	Electrostatic Energy	−18.01	−15.64	−14.88
	Solvation Energy (SE)	11.05	14.93	13.49
	Gas-Phase Energy	−93.05	−88	−80.75
	Total Binding Energy	−82	−73.07	−67.26
MM/PBSA	Van der Waals Energy	−75.04	−72.36	−65.87
	Electrostatic Energy	−18.01	−15.64	−14.88
	Salvation Energy (SE)	13.04	15.82	16.49
	Gas-Phase Energy	−93.05	−88	−80.75
	Total Binding Energy	−80.01	−72.18	−64.26

**Table 5:** The WaterSwap energies for each complex.

Algorithms	PP1γ2-D751-0223	PP1γ2-D751-0143	PP1γ2-N117-0087
Bennet’s	−51.42	−47.50	−37.45
Free-Energy Perturbation (FEP)	−50.24	−46.99	−37.00
Thermodynamic Integration (TI)	−51.49	−47.16	−37.15
Total Mean	−51.05	−47.21	−37.2

## Data Availability

The data generated in the work is presented in the manuscript. All the raw data is also uploaded to the journal along with Supplementary Files.

## Supplementary Materials

The following supporting information can be downloaded at https://www.mdpi.com/article/10.3390/cimb47080658/s1.
