# Supplementary material for: Exploring Novel Inhibitory Compounds Against Phosphatase Gamma 2: A Therapeutic Target for Male Contraceptives
Source: Curr Issues Mol Biol. 2025 Aug 15;47(8):658. doi: 10.3390/cimb47080658 (PMC12384959; doi:10.3390/cimb47080658)
Supplement: Supplementary file 1 [file cimb-47-00658-s001.zip › cimb-3753135-supplementary.pdf]

Supplementary File

Table S1. The pharmacokinetics features of the top three compounds as AZD2281, MDV3100, Carbamazepine, and Control

PHYSIOCHEMICAL PROPERTIES

Compounds	Formula	Molecular weight	Num. heavy atoms	Num. aroma. heavy atoms	Fraction Csp3	Num. H-bond acceptors	Num. -bond donors	Molar Refractivity	TPSA
**D751-0223**	C25H26N6O2S+++	474.58 g/mol	34	20	0.28	2	3	474.58 g/mol	114.10 Å²
**D751-0143**	C27H30N6O3	511.63 g/mc	37	20	0.33	3	3	144.04	102.30 Å²
**N117-0087**	C28H38FN5O3	236.27 g/mol	18	5	0.68	4	0	157.35	67.41 Å²

LIPOPHILICITY

Compounds	Log $P_{o/w}$ (iLOGP)	Log $P_{o/w}$ (XLOGP3)	Log $P_{o/w}$ (WLOGP)	Log $P_{o/w}$ (MLOGP)	Log $P_{o/w}$ (SILICOS-IT)	Consensus Log $P_{o/w}$
**D751-0223**	2.72	2.97	1.54	2.87	3.20	2.66
**D751-0143**	-1.49	4.34	2.57	2.23	2.95	2.12
**N117-0087**	3.60	1.79	1.16	2.85	1.24	2.13

PHARMACOKINETICS

Compounds	GI absorption	BBB permeant	P-gp substrate	CYP1A2 inhibitor	CYP2C19 inhibitor	CYP2C9 inhibitor	CYP2D6 inhibitor	CYP3A4 inhibitor	Log K_p (skin permeation)

**D751-0223**	High	NO	Yes	No	Yes	Yes	Yes	No	-7.09 cm/s
**D751-0143**	High	No	Yes	No	Yes	Yes	Yes	Yes	-6.19cm/s
**N117-0087**	High	Yes	No	No	No	No	Yes	Yes	-8.15 cm/s

DRUG LIKENESS

**Compounds**	**Lipinski**	**Ghose**	**Veber**	**Egan**	**Muegge**	**Bioavailability Score**
**D751-0223**	Yes; 0 Violation	No; 1 violation MR>130	Yes	Yes	Yes	0.55
**D751-0143**	Yes; 0 violation	No; 2 violations: MW>480, MR>130	Yes	Yes	Yes	0.55
**N117-0087**	Yes; 0 violation	Yes	No; 1 violation	Yes	Yes	0.55

MEDICINAL CHEMISTRY

**Compounds**	**PAINS**	**Brenk**	**Lead likeness**	**Synthetic accessibility**
**D751-0223**	1 alert: indol_3yl_alk	0 alert	No, 1 violations	4.30
**D751-0143**	1 alert	0 alerts:	No; 2 violation	4.51
**N117-0087**	0 alert	0 alert:	No; 1 violation:	5.16

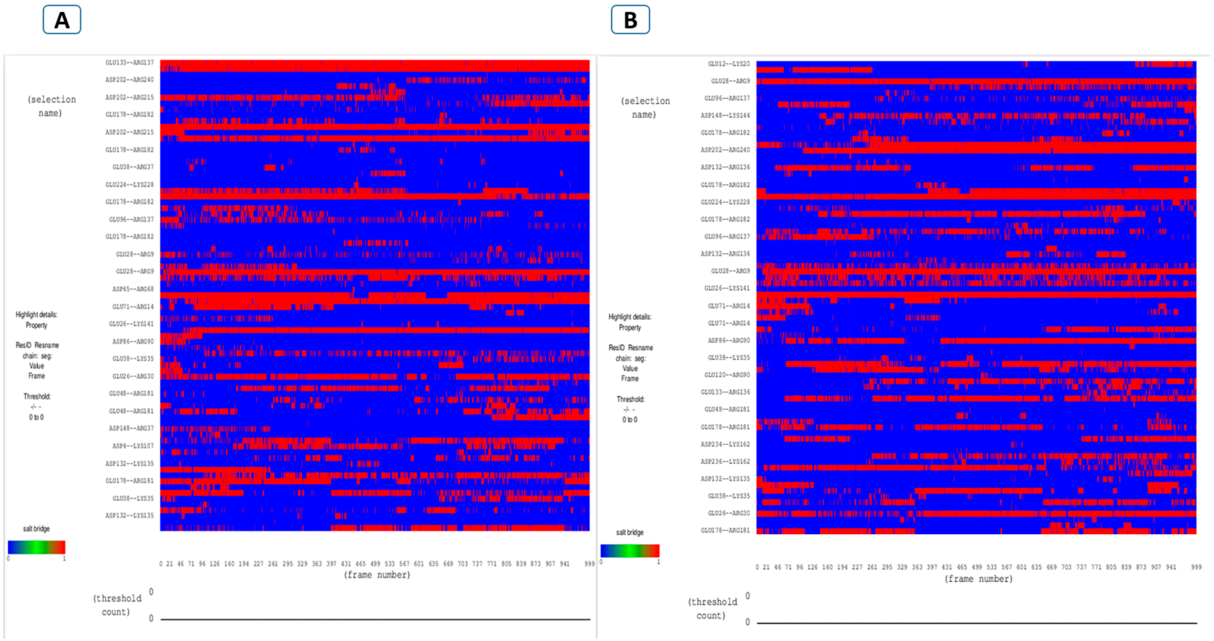

Figure S1.The Salt bridges for **D751-0223** (A) and **D751-0143**(B).

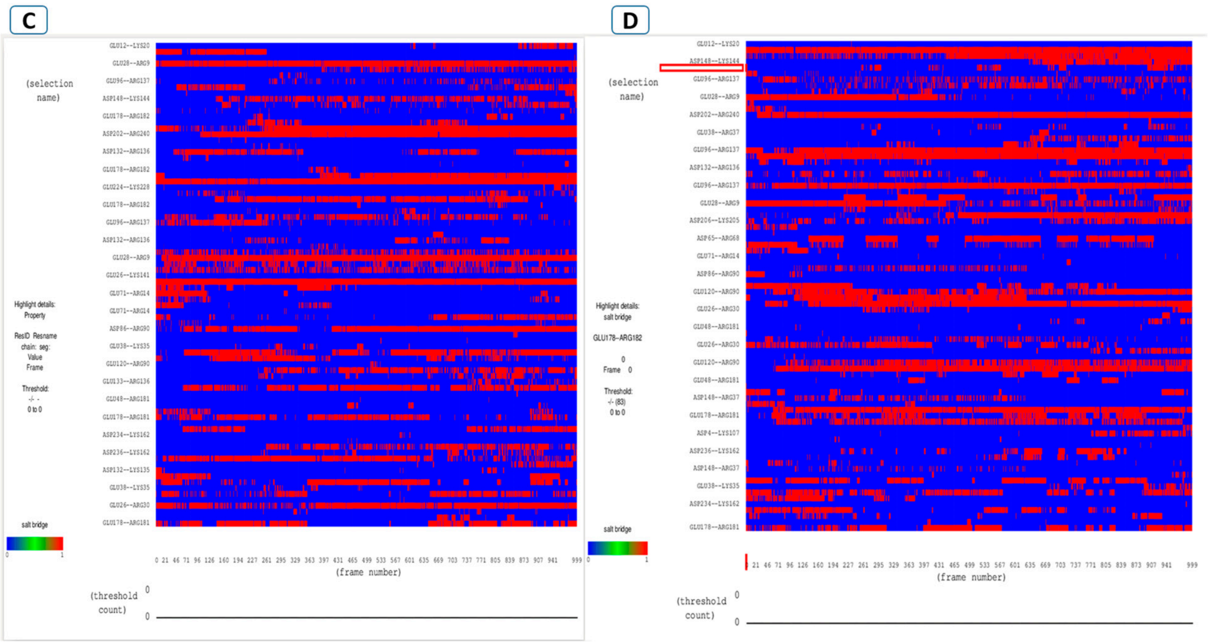

Figure S2: The salt bridges analysis of **N117-0087** (C) and Apo (D)
